# A Population Balance Model to Describe the Evolution of Sublethal Injury

**DOI:** 10.3390/foods10071674

**Published:** 2021-07-20

**Authors:** Simen Akkermans, Davy Verheyen, Cindy Smet, Jan F. M. Van Impe

**Affiliations:** 1 BioTeC, Chemical and Biochemical Process Technology and Control, Department of Chemical Engineering, KU Leuven, 9000 Ghent, Belgium; simen.akkermans@kuleuven.be (S.A.); davy.verheyen@kuleuven.be (D.V.); cindy.smet@kuleuven.be (C.S.); 2 OPTEC, Optimization in Engineering Center-of-Excellence, KU Leuven, 3000 Leuven, Belgium; 3 CPMF2, Flemish Cluster Predictive Microbiology in Foods-www.cpmf2.be, 9000 Ghent, Belgium

**Keywords:** food safety, predictive microbiology, mathematical models, microbial inactivation, sublethal injury

## Abstract

The detection and quantification of sublethal injury (SI) of pathogenic microorganisms has become a common procedure when assessing the efficiency of microbial inactivation treatments. However, while a plethora of studies investigates SI in function of time, no suitable modelling procedure for SI data has been proposed thus far. In this study, a new SI model structure was developed that relies on existing microbial inactivation models. This model is based on the description of inactivation kinetics between the subpopulations of healthy, sublethally injured and dead cells. The model was validated by means of case studies on previously published results, modelled by different inactivation models, i.e., (i) log-linear inactivation; (ii) biphasic inactivation; and (iii) log-linear inactivation with tailing. Results were compared to those obtained by the traditional method that relies on calculating SI from independent inactivation models on non-selective and selective media. The log-linear inactivation case study demonstrated that the SI model is equivalent to the use of independent models when there can be no mistake in calculating SI. The biphasic inactivation case study illustrated how the SI model avoids unrealistic calculations of SI that would otherwise occur. The final case study on log-linear inactivation with tailing clarified that the SI model provides a more mechanistic description than the independent models, in this case allowing the reduction of the number of model parameters. As such, this paper provides a comprehensive overview of the potential and applications for the newly presented SI model.

## 1. Introduction

In predictive microbiology, the effects of intrinsic and/or extrinsic factors on the microbial behaviour (e.g., growth, inactivation) in foods are studied and quantified to predict the effect of varying environmental conditions on the microbial response. Accumulated knowledge on microbial behaviour is distilled into mathematical models, which can be integrated into user-friendly software tools. These tools can be used by food producers, governments and scientists to determine food safety and quality aspects [1,2].

Due to the health consequences related to the ingestion of pathogenic foodborne microorganisms, food industries design processing treatments to inactivate microorganisms that may be present in food products [3]. Exposing food products containing bacterial populations to an inactivation treatment (e.g., heating, irradiation, antimicrobial agents) leads to the formation of three bacterial subpopulations, i.e., (i) healthy cells that are uninjured; (ii) sublethally injured cells that can potentially still recover from their injuries; and (iii) dead cells that are completely inactivated/killed [4]. Sublethal injury (SI) is generally defined as “a consequence of exposure to a chemical or physical process that damages but does not kill a microorganism” [5,6,7]. The injury can involve a loss of the permeability barrier in the cell wall and/or membrane (i.e., structural damage), damage to functional cell components, such as ribosomes and structural DNA (i.e., metabolic damage), or a combination of both types [8]. Sublethally injured cells are sensitive to selective components to which uninjured cells show resistance, making them unable to grow on selective media commonly used for the detection of foodborne pathogens in the food industry [9]. Consequently, the challenges related to the occurrence of sublethally injured cells in foods are twofold. The first challenge relates to food diagnostics, as the number of microorganisms is underestimated when solely enumerated by plating on selective media. As a second challenge, sublethally injured microorganisms might recover from their damage if exposed to optimal conditions for a sufficient amount of time, for example during food storage [10,11,12,13].

The degree of SI of a microbial population is traditionally assessed by the difference in plate counts on non-selective and selective media [7,8,14]. Non-selective media allow the growth of the total population of all viable culturable cells (i.e., uninjured and sublethally injured), while selective media only allow the growth of uninjured cells [15]. Hence, SI can be quantified as a percentage of the entire population by means of formulas similar to Equation (1), introduced by the likes of Busch et al. and Dykes [16,17].

(1) $$\mathrm{SI}=\left(1-\frac{\mathrm{Counts}\;\mathrm{on}\;\mathrm{selective}\;\mathrm{medium}}{\mathrm{Counts}\;\mathrm{on}\;\mathrm{nonselective}\;\mathrm{medium}}\right)\cdot 100\%$$

Following the increased attention to SI in the last decades, the scientific literature experienced a rise in the number of predictive microbiology studies that include a representation of the SI evolution of microorganisms during inactivation. In most studies, SI is calculated at different time points, regardless of the inactivation behaviour of the cells [18,19,20,21,22,23,24,25,26,27,28,29,30,31,32]. To investigate the SI of cells during inactivation treatments in more depth, some researchers have calculated the SI evolution over time based on the modelled inactivation of the total culturable population and the healthy subpopulation. Apart from visually illustrating the SI evolution in a continuous manner, calculating the SI evolution as a function of the inactivation treatment time allows a comparison of total amounts of SI among different treatment conditions, e.g., using the time-averaged injured cells coefficient (TICC) [33]. 

SI cells should not be confused with so-called viable but non-culturable (VBNC) cells. VBNC cells are not regarded as dead cells because their cell membrane and genetic material is intact and they are metabolically active [34]. SI cells are not dead either. They have sustained injuries that cause them not to be viable on stress-containing selective media, but they are still viable on non-selective media. As such, the difference between VBNC and SI cells is that the former cannot be cultured on any media, while the latter can still be cultured on non-selective media [35]. The use of a combination of optimal rich media and selective media therefore allows differentiating between SI cells, on the one hand, and VBNC and dead cells on the other hand. 

**Figure 1:**
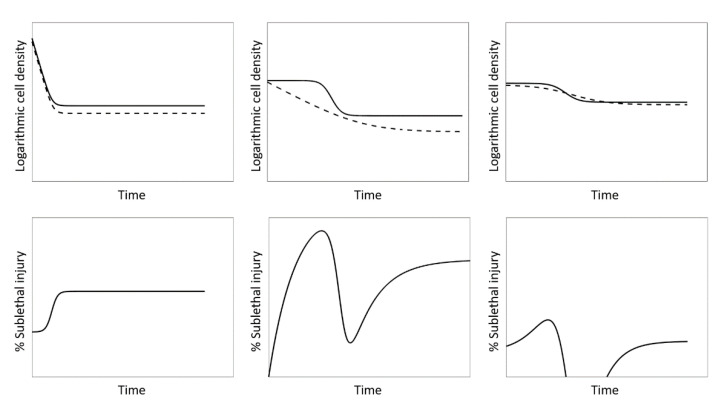
Examples of artefacts occurring due to calculating SI directly from non-log transformed model fit values. The modelled evolution of the population density on non-selective (—) and selective (---) medium is presented in the top row and the resulting SI in the bottom row. Both are presented as a function of unitless time. Left: long periods of constant SI caused by consecutive differences in model fits on the non-selective and selective medium. Middle: strange SI evolution caused by differences in model output shapes on non-selective and selective medium. Right: negative values in the SI evolution caused by intersecting model fits on the non-selective and selective media. For each case, top figures represent the model fits to non-selective and selective media, while bottom figures represent the SI calculated according to Equation (1).

Thus far, different approaches for calculating the SI have been used with no consensus on a standard method having been reached. In the most common method, the SI evolution is calculated by using the non-log transformed model outputs corresponding to non-selective and selective media as input data for the SI equation [36,37,38,39,40,41,42,43]. The major disadvantage related to this methodology is the frequent occurrence of SI trends that are (in part) artefacts of the methodology, rather than accurate representations of physical phenomena, e.g., (i) extended periods with constant values of SI caused by consecutive differences in model fits on the non-selective and selective medium, e.g., during tailing; (ii) strange SI trends caused by differences in the model output behaviour on the non-selective and selective medium; and (iii) negative values of SI caused by intersecting model fits on the non-selective and selective media. Examples of these artefacts are illustrated in Figure 1. Given these artefacts, the methodology requires the assumption of zero SI when the model output on the selective medium is higher than on the non-selective medium. To avoid some of the aforementioned artefacts, Noriega et al. used a slightly different methodology; they directly used the log transformed model fit values on the non-selective and selective medium as input for the SI equation [12]. On the one hand, the adapted approach resulted in more smooth SI evolutions characterised by less abruptly changing SI evolutions. On the other hand, the subpopulation of sublethally injured cells was not represented as a percentage of the total cell population, essentially not corresponding to the SI equation. Moreover, SI evolutions not explained by physical phenomena still occurred due to the SI evolution solely being based on the difference between model fits on non-selective and selective media. Therefore, Verheyen et al. used the raw cell count data directly as an input for the SI equation and fitted a third degree polynomial to the resulting SI data points [44,45]. Log transformed cell count data were used because fitting a model to the absolute values of an exponential process would lead to a large variance in SI values over the course of inactivation treatments. While this methodology was able to describe the SI-behaviour of microbial inactivation fairly well, some artefacts of using the third-degree polynomial as an SI model were observed, e.g., a sudden increase in SI at the end of inactivation treatments. In addition, the subpopulation of sublethally injured cells was not represented as a percentage of the total cell population due to the use of log transformed cell counts, similar to what was the case for the methodology of Noriega et al. [12]. Moreover, this method is a black box approach to describing the evolution of SI, ignoring any mechanistic knowledge that is available on the process. As such, there is also no guarantee that the selected polynomial is appropriate to represent the true underlying phenomena. 

The objective of this study was to develop a SI modelling method specifically tailored to use in combination with existing microbial inactivation models, to avoid disadvantages related to the approaches discussed above. In essence, all these disadvantages can be avoided if a more mechanistic modelling approach is used that can guarantee an appropriate relationship between the model fits to the non-selective and selective media. Therefore, a method was proposed to model the evolution of experimental data both on non-selective and selective media by a single model that describes the relationship between the corresponding populations of all culturable and healthy culturable cells. This methodology assumes that cells are first sublethally injured prior to inactivation; an assumption that can be incorporated directly into primary inactivation models. The newly developed modelling concept was validated by means of three parameter estimation case studies from literature, comparing integrated SI modelling results to the results obtained with the traditional methodology that relies on independent models, as used in the respective papers.

## 2. Materials and Methods

### 2.1. Datasets

Three datasets from previous research by the authors’ research group were used to evaluate the implementation of the novel sublethal injury (SI) model (presented in Section 3.1). For each dataset, the SI model was compared with the classical method of using independent models. The three datasets were selected specifically because they display different inactivation kinetics and therefore require different inactivation models as well. Dataset 1 was published by Smet et al. and displays simple log-linear inactivation kinetics [42]. Specifically, this dataset was obtained by applying cold atmospheric plasma treatments to *Salmonella* typhimurium that was grown as planktonic cells in an environment at pH 5.5 with 60 g/L NaCl. The reported inactivation curves were the result of the storage of samples at 8 °C after they had been treated with this plasma in a liquid carrier. Samples were collected over a period of 770 h and there were 22 samples for both the non-selective medium (TSA; Tryptic Soy Agar; Oxoid, Basingstoke, UK) and selective medium (XLD; Xylose Lysine Deoxycholate agar; Merck & Co, Rahway, NJ, USA). Dataset 2 and Dataset 3 have been published by Noriega et al. in the context of studying the effect of cell immobilization on SI during mild heat treatments (54 °C) [12]. Dataset 2 was from the inactivation of low-density colonies of *Listeria innocua* and displays a biphasic log-linear inactivation. There were 104 datapoints on non-selective medium and 87 on selective medium. The non-selective medium was TSA supplemented with 6 g/L yeast extract (Merck, Darmstadt, Germany) and the selective medium was the same supplemented with an additional 65 g/L NaCl (VWR, Leuven, Belgium). It should be noted that *L. innocua* was used as an innocuous surrogate organism for the food pathogen *L. monocytogenes*. Although this is a commonly used surrogate, it is not as tolerant to stress factors as *L. monocytogenes*. Dataset 3 was obtained from the inactivation of high-density colonies of *Salmonella* Typhimurium and displays log-linear inactivation with a tail. This dataset contained 130 datapoints on non-selective medium (TSA) and 89 datapoints on selective medium (TSA supplemented with 50 g/L NaCl). In this work, all data were converted to be expressed in the natural logarithm instead of the common logarithm of the cell density.

### 2.2. Parameter Estimation and Uncertainty

Model parameters were estimated using the function *lsqnonlin* of MATLAB R2019b (The MathWorks). The parameter estimation was based on the minimization of the sum of squared errors SSE:

(2) $$\mathrm{SSE}=\overset{\nu_{m}}{\underset{i=1}{\sum }}{\left(n_{m,i}\left(t_{i}\right)-n_{p,i}\left(t_{i},p\right)\right)}^{2}\;$$

with $\nu_{m}$ the number of measurements in the dataset, $n_{m,i}$ the logarithm of the measured cell concentration, $n_{p,i}$ the logarithm of the predicted cell concentration, $t_{i}$ the time point corresponding to a specific measurement and p the vector of model parameters. The different models will be described in the results and discussion section when used. The 95% confidence bounds of the estimated parameters were estimated using Equation (3) [46]:

(3) $$\left[{\;p}_{i}\pm t_{0.975,{\;\nu }_{m}-\nu_{p}}\cdot \sigma_{p_{i}}\right]$$

with t as the inverse Student’s t-distribution, $\nu_{p}$ the number of model parameters, and $\sigma_{p_{i}}$ the standard deviation of a model parameter $p_{i}$. This standard deviation was calculated through Equations (4)–(7) [47]:

(4) $$\sigma_{p_{i}}=\sqrt{V\left(i,i\right)}$$

(5) $$V=F^{-1}$$

(6) $$F=\frac{1}{\mathrm{MSE}}\;J^{\prime }\cdot J$$

(7) $$\mathrm{MSE}=\frac{\mathrm{SSE}}{\nu_{m}-\nu_{p}}$$

where V is the variance-covariance matrix of the model parameters, F is the Fisher Information Matrix, J is the Jacobian matrix and MSE is the mean sum of squared errors.

### 2.3. Sublethal Injury

Sublethal injury (SI) is defined here as the percentage of cells that cannot grow on selective media, but are able to grow on non-selective media:

(8) $$\mathrm{SI}=\frac{N_{\mathrm{non}-\mathrm{selective}}-N_{\mathrm{selective}}}{N_{\mathrm{non}-\mathrm{selective}}}\cdot 100\%$$

with $N_{\mathrm{non}-\mathrm{selective}}$ and $N_{\mathrm{selective}}$ the quantity of cells as detected on non-selective and selective media. It should be noted that the actual quantity of cells is used and not a logarithmic transformation of this quantity. As such, SI can be expressed as a percentage. 

## 3. Results and Discussion

The current study presents a new modelling concept to describe sublethal injury (SI) as a function of time through sets of differential equations. A variety of inactivation models is described in literature. Together, these models have the ability to produce a wide variety of inactivation kinetics and they are selected based on the requirements of the specific scenario that is under study. Consequently, the goal of this work is not to present a specific model, but rather to present a method that allows the user to develop a new SI model based on any existing inactivation model that is available as a (set of) differential equation(s). The global concept of this modelling method is unveiled in Section 3.1. Then, this concept is applied to three different scenarios based on different datasets that required different inactivation models (Section 3.2, Section 3.3 and Section 3.4). For each scenario, the SI model is composed, the model parameters to be estimated are identified, the resulting model is tested on an available dataset and the results are compared with what would have been obtained if the data would be treated independently as done in the conventional method. 

### 3.1. The Sublethal Injury Model

**Figure 2:**
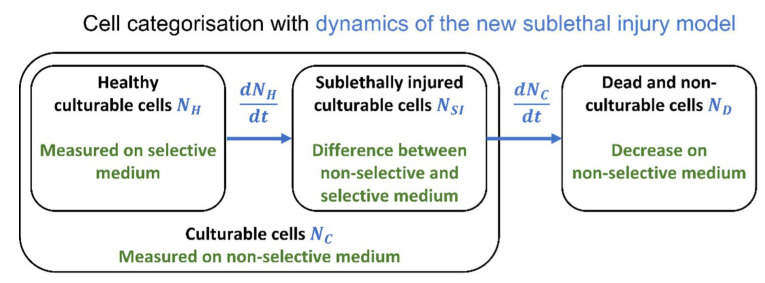
Overview of the microbial subpopulations that are considered in the sublethal injury model. All cells can be divided in culturable and dead cells. The culturable cells can be subdivided in healthy and injured cells. The mathematical notations and conversions are marked in blue and the experimental quantifications are written in green.

The main lack of the conventional approach to describe the evolution of SI is that the evolution of all culturable cells and of the healthy culturable cells as a function of time are treated as independent phenomena. Therefore, the main requirement for the new modelling concept is to link these inactivation kinetics. Composing this model concept is done by defining different subpopulations within the model. The distinct subpopulations are the healthy culturable cells $N_{H}$, the sublethally injured (SI) culturable cells $N_{\mathrm{SI}}$ and the dead (and non-culturable) cells $N_{D}$. Additionally, the healthy cells and injured cells can be grouped together in the class of all culturable cells $N_{C}$. This classification is presented in the Venn diagram of Figure 2. Based on these subpopulations, the inactivation of the microbial population can be described as the transition of cells from one subpopulation to another. A healthy cell can become a SI cell due to the inactivation method or survival conditions that it undergoes. It can be assumed that, under the studied conditions that cause inactivation of the cells, injured cells will be unable to become healthy cells again. Therefore, the rate of cells becoming injured is equal to the change in the quantity of healthy cells. This is described mathematically by a differential equation for ${\mathrm{dN}}_{H}/\mathrm{dt}$. Experimentally, the evolution of the healthy population of cells is monitored through viable plate counting on selective media. Consequently, if injured cells are harmed further, their injuries become irreparable and they are thus converted to dead cells. If one assumes that the healthy cells are always first injured before they can be killed, the rate of injured cells dying off is equal to the change in the population of all culturable cells. This is mathematically expressed by a differential equation for ${\mathrm{dN}}_{L}/\mathrm{dt}$ and the process can be monitored experimentally by using viable plate counts on non-selective medium. This assumption has been confirmed, e.g., in the research of Perni et al. on the inactivation of *E. coli* by pulsed electric fields [48]. If cells first have to become injured before being killed, this also means that the size of ${\mathrm{dN}}_{C}/\mathrm{dt}$ is proportional to the quantity of sublethally injured cells, not to the total quantity of culturable cells. The change of the population of injured cells is then a combination of the transition of healthy cells to injured cells and of injured cells to dead cells (${\mathrm{dN}}_{\mathrm{SI}}/\mathrm{dt}=-{\mathrm{dN}}_{H}/\mathrm{dt}+{\mathrm{dN}}_{C}/\mathrm{dt}$). Within this modelling framework, two differential equations are needed to describe the evolution of the three subpopulations: (i) the change in the number of healthy cells ${\mathrm{dN}}_{H}/\mathrm{dt}$ and (ii) the change in the number of culturable cells ${\mathrm{dN}}_{C}/\mathrm{dt}$. These two populations are monitored experimentally by plating respectively on selective and non-selective medium. As such, it is possible to identify the model equations on the data that are available out of the same types of experiments that are commonly being done to study SI. The data that are already being collected is sufficient, but the model itself needs to link the subpopulations as described above. By linking these subpopulations in a semi-empirical population balance model, there is a guarantee that the model will deliver reasonable predictions of the phenomena where, e.g., the number of healthy cells can never be higher than the total number of culturable cells. To make the proposed modelling concept more tangible, the next three subsections will present how to compose the model equations for the SI model based on three different inactivation models that are taken from literature. 

### 3.2. Case Study 1: Log-Linear Inactivation

The first SI model that is composed here is based on simple log-linear inactivation for both the healthy and sublethally injured populations. As such, the model of Bigelow and Esty can be used to describe the evolution of the healthy population as a function of time [49]:

(9) $$\frac{{\mathrm{dN}}_{H}\left(t\right)}{\mathrm{dt}}=-k_{H,\max}\cdot N_{H}\left(t\right)$$

with $k_{H,\max}$ the maximum specific inactivation rate of the healthy population, which is expressed in an inverse time unit (e.g., 1/min, 1/s). For computational purposes, it is better to work with the logarithmic transformation of the population size:

(10) $$\frac{{\mathrm{dn}}_{H}\left(t\right)}{\mathrm{dt}}=-k_{H,\max}$$

with $n_{H}$ the logarithm of the size of the population of healthy cells. The next equation that can be composed is that for the total population of culturable cells:

(11) $$\frac{{\mathrm{dN}}_{C}\left(t\right)}{\mathrm{dt}}=-k_{\mathrm{SI},\max}\cdot N_{\mathrm{SI}}\left(t\right)$$

with $k_{SI,max}$ the maximum specific inactivation rate of the population of injured cells. This equation demonstrates that the decrease of the total quantity of culturable cells is only a function of the quantity of injured cells. This is a consequence of the assumption that cells first have to become injured before they can be killed. The quantity of injured cells can be calculated as the difference between the total population of culturable cells and the population of healthy cells ($N_{\mathrm{SI}}\left(t\right)=N_{C}\left(t\right)-N_{H}\left(t\right)$). As such, on a logarithmic scale, Equation (11) becomes:

(12) $$\frac{{\mathrm{dn}}_{C}\left(t\right)}{\mathrm{dt}}=-k_{\mathrm{SI},\max}\cdot \left(1-\exp\left(n_{H}\left(t\right)-n_{C}\left(t\right)\right)\right)$$

with $n_{C}$ the logarithm of the size of the population of culturable cells. The differential equation for the evolution of the population of injured cells itself is a combination of the increase due to the healthy cells becoming injured (Equation (9)) and a decrease due to the injured cells dying off (Equation (11)). The Equation (14) for the evolution of the injured population can therefore be composed as:

(13) $$\frac{{\mathrm{dN}}_{\mathrm{SI}}\left(t\right)}{\mathrm{dt}}=-\frac{{\mathrm{dN}}_{H}\left(t\right)}{\mathrm{dt}}+\frac{{\mathrm{dN}}_{C}\left(t\right)}{\mathrm{dt}}$$

(14) $$\frac{{\mathrm{dN}}_{\mathrm{SI}}\left(t\right)}{\mathrm{dt}}=k_{H1,\max}\cdot N_{H}\left(t\right)-k_{\mathrm{SI}1,\max}\cdot N_{\mathrm{SI}}\left(t\right)$$

Converting Equation (14) to the logarithmic scale results in the following equation:

(15) $$\frac{{\mathrm{dn}}_{\mathrm{SI}}\left(t\right)}{\mathrm{dt}}=k_{H1,\max}\cdot \frac{\exp\left(n_{H}\left(t\right)\right)}{\exp\left(n_{\mathrm{SI}}\left(t\right)\right)}-k_{\mathrm{SI},\max}$$

Given (i) that the quantity of injured cells at any point in time can be calculated from the difference between the amount of culturable and healthy cells and (ii) that the experimental data of viable plate counts on non-selective and selective media provides direct information about the quantity of culturable and healthy cells, it is not necessary to include Equation (15) on the evolution of the injured population when solving the differential equations that describe this SI model. Instead, the quantity of injured cells can be calculated from the results of the culturable and healthy populations. As such, the SI model for log-linear inactivation can be described by Equations (10) and (12). To solve this system of differential equations, the initial conditions are required. These initial conditions are:

(16) $$n_{H}\left(t=0\right)=n_{H,0}$$

(17) $$n_{C}\left(t=0\right)=n_{C,0}$$

with $n_{H,0}$ and $n_{C,0}$ the logarithm of the population sizes at the initial time point. These initial conditions cannot take any value as the initial quantity of all culturable cells should of course be larger than its subpopulation of initial healthy cells. This constraint can be added into the model by converting the first initial condition to:

(18) $$n_{H}\left(t=0\right)=f_{H,0}\cdot n_{C,0}\;\mathrm{with}\;f_{H,0}\;\epsilon \;\left[0;1\right]$$

with $f_{H,0}$ the fraction of the total culturable population of cells that are healthy cells. The full model can thus be summarised as:

(19) $$\frac{{\mathrm{dn}}_{H}\left(t\right)}{\mathrm{dt}}=-k_{H,\max}\;\mathrm{with}\;n_{H}\left(t=0\right)=f_{H,0}\cdot n_{C,0}\;\mathrm{and}\;f_{H,0}\;\epsilon \;\left[0;1\right]$$

(20) $$\frac{{\mathrm{dn}}_{C}\left(t\right)}{\mathrm{dt}}=-k_{\mathrm{SI},\max}\cdot \left(1-\exp\left(n_{H}\left(t\right)-n_{C}\left(t\right)\right)\right)\;\mathrm{with}\;n_{C}\left(t=0\right)=n_{L,0}$$

with the following model parameters to be estimated: $f_{H,0}$, $n_{C,0}$, $k_{H,\max}$ and $k_{\mathrm{SI},\max}$. In contrast, when considering the healthy and culturable populations as independent populations, they would be described by the following independent models:

(21) $$\frac{{\mathrm{dn}}_{H}\left(t\right)}{\mathrm{dt}}=-k_{H,\max}\;\mathrm{with}\;n_{H}\left(t=0\right)=n_{H,0}$$

(22) $$\frac{{\mathrm{dn}}_{C}\left(t\right)}{\mathrm{dt}}=-k_{C,\max}\;\mathrm{with}\;n_{C}\left(t=0\right)=n_{C,0}$$

where $k_{C,\max}$ is the maximum specific inactivation rate of the population of culturable cells. The model parameters in these independent models are $n_{H,0}$, $n_{C,0}$, $k_{H,\max}$ and $k_{C,\max}$.

**Figure 3:**
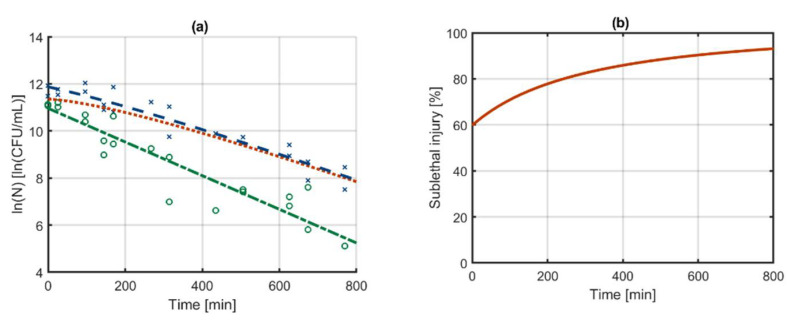
(**a**) Log-linear inactivation of all culturable (---) and healthy culturable (-·-) cells as described by the independent models or sublethal injury model that is constructed with the model of Bigelow and Esty [49]. Based on these modelling results, the evolution of the data are sublethally injured population (···) and (**b**) the percentage sublethal injury (**—**) is calculated. The based on counts of culturable (x) and healthy (o) cells on non-selective and selective media as published in Smet et al. [42]. The dataset was constructed by monitoring the survival of *Salmonella* Typhimurium, which was grown as planktonic cells in an environment at pH 5.5 with 60 g/L NaCl, after being treated in a liquid carrier with cold atmospheric plasma and stored at 8 °C (Dataset 1).

The SI model of Equations (19) and (20) is compared with the independent model of Equations (21) and (22) based on Dataset 1 (see Section 2.1). For this comparison, the model parameters of each set of equations were estimated and the results are presented in Table 1. The parameters $n_{C,0}$ and $k_{H,\max}$, which appear in both models, have identical values and confidence bounds. The parameters $k_{\mathrm{SI},\max}$ and $k_{C,\max}$ of respectively the SI model and the independent models also have identical values and confidence bounds. These parameters are essentially each other’s equivalent for the dataset that was used in this example. In contrast, the initial number of cells in the independent models is described by the parameter $n_{H,0}$, whereas for the SI model the parameter $f_{H,0}$ determines the fraction of the $n_{C,0}$ that are healthy cells. Relative to their sizes, the uncertainty on the parameter $f_{H,0}$ appears to be high compared to that of $n_{H,0}$. However, the parameter $f_{H,0}$ expresses the fraction of the population size on a linear scale whereas the parameter $n_{H,0}$ follows a logarithmic scale. On the other hand, there is indeed additional uncertainty in the calculation of the initial population of healthy cells in the SI model because its value depends on both $f_{H,0}$ and $n_{C,0}$, which are both marked by uncertainty. The model predictions of the SI model and the independent models are visually indistinguishable and are therefore both represented by the graphs in Figure 3a. The sublethal injury (SI) is calculated as a function of time based on the following equation:

(23) $$\mathrm{SI}\left(t\right)=\frac{\exp\left(n_{C}\left(t\right)\right)-\exp\left(n_{H}\left(t\right)\right)}{\exp\left(n_{C}\left(t\right)\right)}$$

The evolution of SI with time as calculated by both models is illustrated in Figure 3b.

This comparison of the model parameters and predictions of the SI model and independent models based on Dataset 1 demonstrates that the SI model is in a way equivalent to the independent models in cases where there can be no mistake in the calculation of the SI. Even though the equations and the model parameter constraints of both models are different, the model predictions are quasi-identical and the model parameters are equivalent. As will be demonstrated in the next section, these models start to differ when the independent model would yield wrongful predictions of the SI.

### 3.3. Case Study 2: Biphasic Inactivation

**Figure 4:**
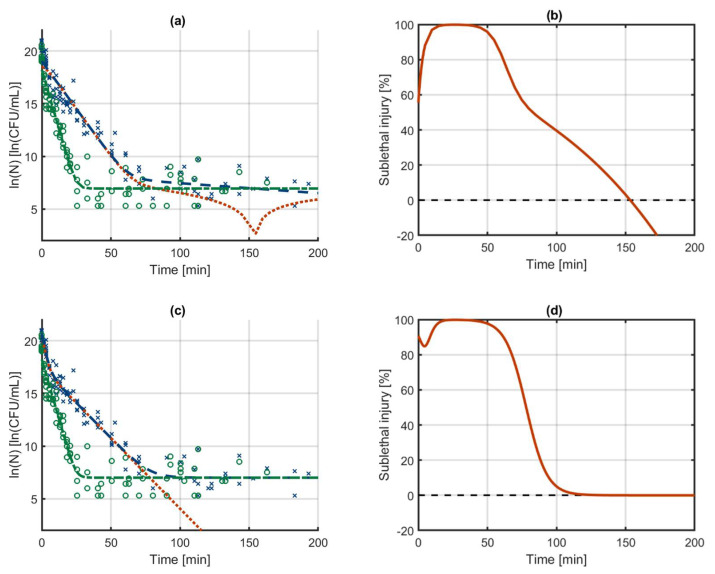
Biphasic inactivation of all culturable (---) and healthy culturable (-·-) cells as described by (**a**) the independent models and (**c**) the sublethal injury model that are constructed with the model of Cerf [50]. Based on these modelling results, the evolution of the sublethally injured population (···) and the percentage sublethal injury (—, (**b**,**d**) are calculated. The data are based on counts of culturable (x) and healthy (o) cells on non-selective and selective media as published by Noriega et al. [12]. The data were obtained by heat-treating low-density colonies of *Listeria innocua* at 54 °C (Dataset 2).

Dataset 2 portrays inactivation data that follows biphasic inactivation kinetics, as can be seen from Figure 4. Biphasic microbial inactivation is typically modelled with the model of Cerf [50]. Independent models for the healthy and culturable cells can be defined based on the model of Cerf as follows:

(24) $$\frac{{\mathrm{dn}}_{H1}\left(t\right)}{\mathrm{dt}}=-k_{H1,\max}\;\mathrm{with}\;n_{H1}\left(t=0\right)=n_{H1,0}$$

(25) $$\frac{{\mathrm{dn}}_{H2}\left(t\right)}{\mathrm{dt}}=-k_{H2,\max}\;\mathrm{with}\;n_{H2}\left(t=0\right)=n_{H2,0}$$

(26) $$\frac{{\mathrm{dn}}_{C1}\left(t\right)}{\mathrm{dt}}=-k_{C1,\max}\;\mathrm{with}\;n_{C1}\left(t=0\right)=n_{C1,0}$$

(27) $$\frac{{\mathrm{dn}}_{C2}\left(t\right)}{\mathrm{dt}}=-k_{C2,\max}\;\mathrm{with}\;n_{C2}\left(t=0\right)=n_{C2,0}$$

with $n_{H1}$ and $n_{H2}$ the healthy cells and $n_{C1}$ and $n_{C2}$ the culturable cells of, respectively, the first and second subpopulation. If one subpopulation has both a lower initial size and a lower inactivation rate than the other, the typical biphasic inactivation is observed [51]. The parameters of the independent models that have to be estimated are $n_{H1,0}$, $n_{H2,0}$, $n_{C1,0}$, $n_{C2,0}$, $k_{H1,\max}$, $k_{H2,\max}$, $k_{C1,\max}$, and $k_{C2,\max}$. When constructing the SI model based on the model of Cerf, the only change in the equations for the evolution of healthy cells in each subpopulation compared to Equations (24) and (25) is that the initial number of cells is defined as a fraction of the initial number of total culturable cells in the respective subpopulation ($f_{H1,0}$ and $f_{H2,0}$):

(28) $$\frac{{\mathrm{dn}}_{H1}\left(t\right)}{\mathrm{dt}}=-k_{H1,\max}\;\mathrm{with}\;n_{H1}\left(t=0\right)=f_{H1,0}\cdot n_{C1,0}\;\mathrm{and}\;f_{H1,0}\;\epsilon \;\left[0;1\right]$$

(29) $$\frac{{\mathrm{dn}}_{H2}\left(t\right)}{\mathrm{dt}}=-k_{H2,\max}\;\mathrm{with}\;n_{H2}\left(t=0\right)=f_{H2,0}\cdot n_{C2,0}\;\mathrm{and}\;f_{H2,0}\;\epsilon \;\left[0;1\right]$$

The differential equations for the two subpopulations of culturable cells are analogous to the differential equation for the culturable cells in the SI model for log-linear inactivation (Equation (20)):

(30) $$\frac{{\mathrm{dn}}_{C1}\left(t\right)}{\mathrm{dt}}=-k_{\mathrm{SI}1,\max}\cdot \left(1-\exp\left(n_{H1}\left(t\right)-n_{C1}\left(t\right)\right)\right)\;\mathrm{with}\;n_{C1}\left(t=0\right)=n_{C1,0}$$

(31) $$\frac{{\mathrm{dn}}_{C2}\left(t\right)}{\mathrm{dt}}=-k_{\mathrm{SI}2,\max}\cdot \left(1-\exp\left(n_{H2}\left(t\right)-n_{C2}\left(t\right)\right)\right)\;\mathrm{with}\;n_{C2}\left(t=0\right)=n_{\mathrm{LC},0}$$

The model parameters of the SI model in Equations (28)–(31) are $f_{H1,0}$, $f_{H2,0}$, $n_{C1,0}$, $n_{C2,0}$, $k_{H1,\max}$, $k_{H2,\max}$, $k_{C1,\max}$ and $k_{C2,\max}$. When comparing the model structure and parameterization between the two models, the differences in the SI model (Equations (28)–(31)) compared to the independent model (Equations (23)–(27)) are (i) that the initial condition of the healthy subpopulation is defined as a fraction of the initial culturable subpopulation and (ii) that the inactivation rate of the culturable cells depends on the number of injured cells instead of on the number of culturable cells. 

Both models were fitted on the Dataset 2. The resulting model outputs and the calculated SI evolution are illustrated in Figure 4. In the results for the independent models, the model output for the culturable cells decreases below the model output for the healthy cells. In practice, it is of course not possible that the number of healthy cells would ever be higher than the number of culturable cells since the former is a subpopulation of the latter. However, due to this error, the number of injured cells that is calculated becomes negative and its logarithm a complex number. This is all because the independent models do not consider that the data are meant to describe the phenomenon of SI. The model output of the SI model on the other hand provides a good approximation of the experimental data and a reasonable calculation of the evolution of SI. The parameter estimation results for the independent models in Table 2 do not reveal the problems that occur when calculating the SI. Since the independent models fit the respective datasets for culturable and healthy cells well, the model parameter uncertainty is relatively low and the model fit is decent (as seen from the MSE). The problems in this approach only occurs when using the model outputs for calculating the SI. When comparing the parameters of the independent models and the SI model, the large difference in the initial populations catches the eye. These values of the initial populations are part of predicting the biphasic behaviour. In the SI model, the decrease of the culturable population is bound by the number of healthy cells. As such, there is no need for the biphasic behaviour of the inactivation model to predict the levelling off of the culturable cells towards the healthy cells. Consequently, when fitting the model parameters of the SI, the flexibility of the biphasic model is used to predict faster inactivation in the first 10 h compared to the time interval between 10 and 70 h (see Figure 4c). This phenomenon can also be observed from comparing the inactivation rate of the culturable population at time zero in the SI model (as determined by $k_{\mathrm{SI}2,\max}$) with that of the independent models (as determined by $k_{C2,\max}$). This comparison shows that the initial inactivation rate in the SI model is much higher than in the independent models. These differences in the model structure also resulted in a better model fit of the SI model, as indicated from the MSE. Due to its constraints, the SI model will not always provide a better approximation of experimental data than the independent models. On the other hand, these constraints cause the SI model to be able to describe some phenomena with less model complexity than the independent models. As an example, in the current modelling results for the SI model, the tail in the culturable population of cells is a result from the tail in the healthy population of cells. As such, this tailing is described by the model without the need for it to be included separately in the differential equations of the culturable population of cells. In this case, the additional model complexity of the two phases is used to obtain a better approximation of the microbial inactivation in the initial stage of the experiment. The next section will further demonstrate the possibility to work with a decreased model complexity in the SI model.

This example demonstrates that the proposed SI model provides a guarantee for reasonable predictions on the evolution of SI by incorporating basic knowledge on the relationship of the different microbial subpopulation into the model structure.

### 3.4. Case Study 3: Log-Linear Inactivation with Tailing

**Figure 5:**
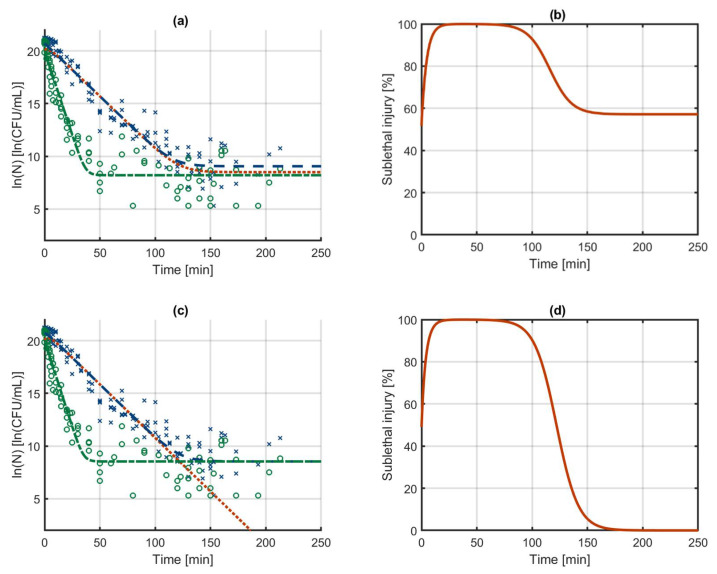
Log-linear inactivation with tailing of all culturable (---) and healthy culturable (-·-) cells as described by (**a**) the independent models and (**c**) the sublethal injury model that are constructed with the model of Geeraerd et al. [52]. Based on these modelling results, the evolution of the sublethally injured population (···) and the percentage sublethal injury (—), (**b**,**d**) are calculated. The data are based on counts of culturable (x) and healthy (o) cells on non-selective and selective media as published by Noriega et al. [12]. The data were obtained by heat-treating high-density colonies of *Salmonella* Typhimurium at 54 °C (Dataset 3).

Figure 5a illustrates the log-linear inactivation behaviour with tailing of both the population of culturable cells and the subpopulation of healthy cells in Dataset 3. Based on the inactivation model of Geeraerd et al., the following independent models are proposed to describe the populations of culturable and healthy cells [52]:

(32) $$\frac{{\mathrm{dn}}_{H\left(t\right)}}{\mathrm{dt}}=k_{H,\max}\cdot \left(1-\exp\left(n_{H,\mathrm{res}}-n_{H}\left(t\right)\right)\right)\;\mathrm{with}\;n_{H}\left(t=0\right)=n_{H,0}$$

(33) $$\frac{{\mathrm{dn}}_{C\left(t\right)}}{\mathrm{dt}}=k_{C,\max}\cdot \left(1-\exp\left(n_{C,\mathrm{res}}-n_{C}\left(t\right)\right)\right)\;\mathrm{with}\;n_{C}\left(t=0\right)=n_{C,0}$$

with $n_{H,\mathrm{res}}$ and $n_{C,\mathrm{res}}$ the resistant subpopulations of the healthy and culturable cells. Converting these models into a linked SI model, results in the following set of equations:

(34) $$\frac{{\mathrm{dn}}_{H\left(t\right)}}{\mathrm{dt}}=k_{H,\max}\cdot \left(1-\exp\left(n_{H,\mathrm{res}}-n_{H}\left(t\right)\right)\right)$$

(35) $$\frac{{\mathrm{dn}}_{C\left(t\right)}}{\mathrm{dt}}=k_{\mathrm{SI},\max}\cdot \left(1-\frac{\exp\left(n_{\mathrm{SI},\mathrm{res}}\right)}{\exp\left(n_{\mathrm{SI}}\left(t\right)\right)}\right)\cdot \frac{\exp\left(n_{\mathrm{SI}}\left(t\right)\right)}{\exp\left(n_{C}\left(t\right)\right)}$$

with $n_{\mathrm{SI},\mathrm{res}}$ the resistant population of injured cells. To obtain a set of differential equations that is only a function of the healthy and culturable cells, Equation (35) is rewritten to:

(36) $$\frac{{\mathrm{dn}}_{C\left(t\right)}}{\mathrm{dt}}=k_{I,\max}\cdot \left(1-\frac{\exp\left(n_{C,\mathrm{res}}\right)-\exp\left(n_{H,\mathrm{res}}\right)}{\exp\left(n_{C}\left(t\right)\right)-\exp\left(n_{H}\left(t\right)\right)}\right)\cdot \frac{\exp\left(n_{C}\left(t\right)\right)-\exp\left(n_{C}\left(t\right)\right)}{\exp\left(n_{C}\left(t\right)\right)}$$

If the model in Equation (36) is used, the resistant population of healthy cells needs to be described as a fraction of the resistant population of culturable cells $f_{H,\mathrm{res}}$:

(37) $$n_{H,\mathrm{res}}=f_{H,\mathrm{res}}\cdot n_{C,\mathrm{res}}$$

When assuming all cells that can become injured can also be killed, there would be no residual population of injured cells. Consequently, the SI model for log-linear inactivation with tailing could be written as:

(38) $$\frac{{\mathrm{dn}}_{H\left(t\right)}}{\mathrm{dt}}=k_{H,\max}\cdot (1-\exp\left(n_{H,\mathrm{res}}-n_{H}\left(t\right)\right))\;\mathrm{with}\;n_{H}\left(t=0\right)=n_{H0}$$

(39) $$\frac{{\mathrm{dn}}_{C\left(t\right)}}{\mathrm{dt}}=k_{\mathrm{SI},\max}\cdot (1-\exp\left(n_{H}\left(t\right)-n_{C}\left(t\right)\right))\;\mathrm{with}\;n_{C}\left(t=0\right)=n_{C0}$$

These two equations basically describe log-linear inactivation with tailing for the subpopulation of healthy culturable cells, while the decrease of the total culturable population depends on the log-linear inactivation of the injured subpopulation. In this model, the decrease of the population of culturable cells will asymptotically approach the resistant population of healthy cells. The independent models of Equations (32) and (33) and the SI model of Equations (38) and (39) are fitted to Dataset 3. The model outputs of both models and the calculated SI are presented in Figure 5. For the independent models, the resistant population of the culturable cells is slightly higher than that of the healthy cells, suggesting that a fraction of the injured cells would be resistant as well. On the other hand, according to the SI model, the total population of culturable cells approaches the resistant population of healthy cells under the assumption that all injured cells are killed. Based on the current data and modelling results, there is of course no way of telling which model predicts the correct inactivation of each subpopulation. However, it would be perfectly possible to use the extended SI model of Equations (34) and (36), which would give practically the same model output as the simplified model of Equations (38) and (39). Because the estimation of the resistant population often comes with quite some uncertainty, it often happens that the resistant population of the culturable cells drops below that of the healthy cells, resulting in a negative SI percentage (similar to Case study 2). This problem will in any case be avoided when using the SI model that is presented here. The model parameters that are estimated are listed in Table 3. For the main part, the parameters are again equivalent between both models, with the main difference that the SI model now has one parameter less than the independent models. Even though the number of model parameters is reduced from six to five, the model accuracy only reduces slightly as shown by the MSE that changes from 1.59 to 1.63.

## 4. Conclusions

A new method was proposed to model the evolution of sublethal injury (SI) during microbial inactivation. This method relies on defining the two subpopulations of healthy and injured cells within the culturable cells and describing inactivation as a two-step mechanism: (i) injuring healthy cells and (ii) killing injured cells. Based on three case studies, it was illustrated that this model concept can be applied to any existing dynamic inactivation model that is available in literature. Moreover, these case studies demonstrated, respectively, three important properties of the SI model: (i) the model is equivalent to the conventional method of using independent models when there is sufficient difference between the total quantity of culturable cells and the subpopulation of healthy cells, i.e., when there can be no mistake in the calculated SI; (ii) the new modelling method avoids unrealistic calculations of the SI by using its mechanistic definition of this process; and (iii) in some cases, it is possible to reduce the number of model parameters in the SI model because there is no need for additional model parameters to describe phenomena that are already hard coded into the more mechanistic description of the model. These properties make the SI model suitable for describing SI as a function of time during food processes. As such, this model can contribute to the optimization and control of the safety of food processes by considering SI dynamics. 

## Figures and Tables

**Table 1:** Parameter estimation results from fitting the independent models (Equations (21) and (22)) and sublethal injury model (Equations (19) and (20)) based on log-linear inactivation on Dataset 1.

Model Parameter	Parameter Estimate	95% Confidence Bounds
Independent models		
$n_{H,0}$	11.0	[10.6; 11.4]
$n_{C,0}$	11.9	[11.4; 12.3]
$k_{H,\max}$	$7.15\times 10^{-3}$	$\left[6.19\times 10^{-3};\;8.11\times 10^{-3}\right]$
$k_{C,\max}$	$5.95\times 10^{-3}$	$\left[4.74\times 10^{-3};\;7.15\times 10^{-3}\right]$
MSE	0.344	
Sublethal injury model		
$f_{H,0}$	0.402	[0.143; 0.662]
$n_{C,0}$	11.9	[11.4; 12.3]
$k_{H,\max}$	$7.15\times 10^{-3}$	$\left[6.19\times 10^{-3};\;8.11\times 10^{-3}\right]$
$k_{\mathrm{SI},\max}$	$5.95\times 10^{-3}$	$\left[4.74\times 10^{-3};\;7.15\times 10^{-3}\right]$
MSE	0.344	

**Table 2:** Parameter estimation results from fitting the independent models (Equations (24) to (27)) and sublethal injury model (Equations (28) to (31)) based on biphasic inactivation on Dataset 2.

Model Parameter	Parameter Estimate	95% Confidence Bounds
Independent models		
$n_{H1,0}$	18.2	[17.7; 18.7]
$n_{H2,0}$	6.96	[5.96; 7.96]
$n_{C1,0}$	8.40	[6.64; 10.2]
$n_{C2,0}$	19.0	[18.6; 19.4]
$k_{H1,\max}$	$4.52\times 10^{-1}$	$\left[4.03\times 10^{-1};\;5.02\times 10^{-1}\right]$
$k_{H2,\max}$	$2.34\times 10^{-14}$	$\left[-1.15\times 10^{-2};\;1.15\times 10^{-2}\right]$
$k_{C1,\max}$	$9.41\times 10^{-3}$	$\left[-4.50\times 10^{-3};\;2.33\times 10^{-2}\right]$
$k_{C2,\max}$	$1.80\times 10^{-1}$	$\left[1.62\times 10^{-1};\;1.99\times 10^{-1}\right]$
MSE	1.41	
Sublethal injury model		
$f_{H1,0}$	$2.91\times 10^{-5}$	$\left[1.17\times 10^{-6};5.71\times 10^{-5}\right]$
$f_{H2,0}$	$9.62\times 10^{-2}$	$\left[1.44\times 10^{-2};\;1.78\times 10^{-1}\right]$
$n_{C1,0}$	17.5	[16.8; 18.1]
$n_{C2,0}$	20.6	[19.8; 21.3]
$k_{H1,\max}$	$1.09\times 10^{-10}$	$\left[-7.16\times 10^{-3};\;7.16\times 10^{-3}\right]$
$k_{H2,\max}$	$4.53\times 10^{-1}$	$\left[4.09\times 10^{-1};\;4.97\times 10^{-1}\right]$
$k_{\mathrm{SI}1,\max}$	$1.34\times 10^{-1}$	$\left[1.15\times 10^{-1};\;1.52\times 10^{-1}\right]$
$k_{\mathrm{SI}2,\max}$	$7.48\times 10^{-1}$	$\left[3.59\times 10^{-1};\;1.14\right]$
MSE	1.14	

**Table 3:** Parameter estimation results from fitting the independent models (Equations (32) and (33)) and sublethal injury model (Equations (38) and (39)) based on log-linear inactivation with tailing on Dataset 3.

Model Parameter	Parameter Estimate	95% Confidence Bounds
Independent models		
$n_{H,0}$	20.0	[19.5; 20.5]
$n_{C,0}$	20.7	[20.3; 21.1]
$n_{H,\mathrm{res}}$	8.21	[7.81; 8.61]
$n_{C,\mathrm{res}}$	9.06	[8.56; 9.56]
$k_{H,\max}$	$3.19\times 10^{-1}$	$\left[2.79\times 10^{-1};\;3.59\times 10^{-1}\right]$
$k_{C,\max}$	$1.01\times 10^{-1}$	$\left[9.28\times 10^{-1};\;1.08\times 10^{-1}\right]$
MSE	1.59	
Sublethal injury model		
$f_{H,0}$	$9.67\cdot 10^{-1}$	$\left[9.32\times 10^{-1};\;1.00\right]$
$n_{C,0}$	20.7	[20.3; 21.2]
$n_{H,\mathrm{res}}$	8.54	[8.22; 8.86]
$k_{H,\max}$	$3.29\times 10^{-1}$	$\left[2.87\times 10^{-1};\;3.72\times 10^{-1}\right]$
$k_{\mathrm{SI},\max}$	$1.02\times 10^{-1}$	$\left[9.46\times 10^{-2};\;1.09\times 10^{-1}\right]$
MSE	1.63	

## Data Availability

All data is available without restrictions upon request through contact with the corresponding author.

## References

[1] McMeekin T.A., Olley J., Ratkowsky D.A., Ross T. (2002). Predictive microbiology: Towards the interface and beyond. Int. J. Food Microbiol..

[2] Pérez-Rodríguez F., Carrasco E., Pradhan A.K., Sant’Ana A.S., Valdramidis V.P., Valero A. (2019). Special issue on 10th international conference of predictive modelling in foods: Towards a new paradigm in predictive microbiology. Int. J. Food Microbiol..

[3] Zwietering M.H., Garre A., Wiedmann M., Buchanan R.L. (2021). All food processes have a residual risk, some are small, some very small and some are extremely small: Zero risk does not exist. Curr. Opin. Food Sci..

[4] Wu V.C.H., Fung D.Y.C. (2001). Evaluation of Thin Agar Layer Method for Recovery of Heat-Injured Foodborne Pathogens. J. Food Sci..

[5] Hurst A. (1977). Bacterial injury: A review. Can. J. Microbiol..

[6] Russell A.D., Andrew M.H.E., Russell A.D. (1984). Potential sites of damage in microorganisms exposed to chemical or physical agents. The Revival of Injured Microbes.

[7] Wesche A.M., Gurtler J.B., Marks B.P., Ryser E.T. (2009). Stress, Sublethal Injury, Resuscitation, and Virulence of Bacterial Foodborne Pathogens. J. Food Prot..

[8] Brashears M.M., Amezquita A., Stratton J. (2001). Validation of Methods Used To Recover *Escherichia coli* O157:H7 and *Salmonella* spp. Subjected to Stress Conditions. J. Food Prot..

[9] Jasson V., Rajkovic A., Debevere J., Uyttendaele M. (2009). Kinetics of resuscitation and growth of *L. monocytogenes* as a tool to select appropriate enrichment conditions as a prior step to rapid detection methods. Food Microbiol..

[10] Deisingh A.K., Thompson M. (2004). Strategies for the detection of *Escherichia coli* O157: H7 in foods. J. Appl. Microbiol..

[11] Jasson V., Uyttendaele M., Raykovic A., Debevere J. (2007). Establishment of procedures provoking sub-lethal injury of *Listeria monocytogenes*, *Campylobacter jejuni* and *Escherichia coli* O157 to serve method performance testing. Int. J. Food Microbiol..

[12] Noriega E., Velliou E.G., Van Derlinden E., Mertens L., Van Impe J.F. (2013). Effect of cell immobilization on heat-induced sublethal injury of *Escherichia coli*, *Salmonella Typhimurium* and *Listeria innocua*. Food Microbiol..

[13] Vermeiren L., Devlieghere F., Vandekinderen I., Rajtak U., Debevere J. (2006). The sensory acceptability of cooked meat products treated with a protective culture depends on glucose content and buffering capacity: A case study with *Lactobacillu sakei* 10A. Meat Sci..

[14] Semanchek J.J., Golden D.A. (1998). Influence of growth temperature on inactivation and injury of *Escherichia coli* O157: H7 by heat, acid, and freezing. J. Food Prot..

[15] Wu V.C.H. (2008). A review of microbial injury and recovery methods in food. Food Microbiol..

[16] Busch S.V., Donnelly C.W. (1992). Development of a Repair-Enrichment Broth for Resuscitation of Heat-Injured *Listeria monocytogenes* and *Listeria innocua*. Appl. Environ. Microbiol..

[17] Dykes G.A. (1999). Physical and metabolic causes of sub-lethal damage in Listeria monocytogenes after long-term chilled storage at 4 °C. J. Appl. Microbiol..

[18] Bi X., Wang Y., Zhao F., Sun Z., Hu X., Liao X. (2015). Sublethal injury and recovery of *Escherichia coli* O157:H7 by high pressure carbon dioxide. Food Control.

[19] Carroll L.M., Bergholz T.M., Hildebrandt I.M., Marks B.P. (2016). Application of a Nonlinear Model to Transcript Levels of Upregulated Stress Response Gene ipbA in Stationary-Phase *Salmonella enterica* Subjected to Sublethal Heat Stress. J. Food Prot..

[20] Ghate V., Leong A.L., Kumar A., Bang W.S., Zhou W., Yuk H.-G. (2015). Enhancing the antibacterial effect of 461 and 521 nm light emitting diodes on selected foodborne pathogens in trypticase soy broth by acidic and alkaline pH conditions. Food Microbiol..

[21] Huang M., Zhuang H., Wang J., Yan W., Zhao J., Zhang J. (2018). Inactivation Kinetics of *Salmonella* Typhimurium and *Staphylococcus aureus* in Different Media by Dielectric Barrier Discharge Non-Thermal Plasma. Appl. Sci..

[22] Lv R., Wang D., Zou M., Wang W., Ma X., Chen W., Zhou J., Ding T., Ye X., Liu D. (2019). Analysis of *Bacillus cereus* cell viability, sublethal injury, and death induced by mild thermal treatment. J. Food Saf..

[23] Olszewska M.A., Zhao T., Doyle M.P. (2016). Inactivation and induction of sublethal injury of *Listeria monocytogenes* in biofilm treated with various sanitizers. Food Control.

[24] Pan Y., Cheng J.-H., Lv X., Sun D.-W. (2019). Assessing the inactivation efficiency of Ar/O2 plasma treatment against *Listeria monocytogenes* cells: Sublethal injury and inactivation kinetics. LWT Food Sci. Technol..

[25] Sanz-Puig M., Moreno P., Pina-Pérez M.C., Rodrigo D., Martínez A. (2017). Combined effect of high hydrostatic pressure (HHP) and antimicrobial from agro-industrial by-products against S. Typhimurium. LWT Food Sci. Technol..

[26] Shao L., Liu Y., Tian X., Wang H., Yu Q., Li X., Dai R. (2020). Inactivation of *Staphylococcus aureus* in phosphate buffered saline and physiological saline ohmic heating with different voltage gradient and frequency. J. Food Eng..

[27] Shi H., Chen Z., Chen D., Kan J. (2017). Sublethal injury and recovery of *Escherichia coli* O157:H7 and K-12 after exposure to lactic acid. Food Control.

[28] Silva A., Genovés S., Martorell P., Zanini S.F., Rodrigo D., Martinez A. (2015). Sublethal injury and virulence changes in *Listeria monocytogenes* and *Listeria innocua* treated with antimicrobials carvacrol and citral. Food Microbiol..

[29] Thomas-Popo E., Mendonca A., Dickson J., Shaw A., Coleman S., Daraba A., Jackson-Davis A., Woods F. (2019). Isoeugenol significantly inactivates *Escherichia coli* O157:H7, *Salmonella enterica*, and *Listeria monocytogenes* in refrigerated tyndallized pineapple juice with added *Yucca schidigera* extract. Food Control.

[30] Wang X., Devlieghere F., Geeraerd A., Uyttendaele M. (2017). Thermal inactivation and sublethal injury kinetics of *Salmonella enterica* and *Listeria monocytogenes* in broth versus agar surface. Int. J. Food Microbiol..

[31] Zhang H., Zhao Y., Gong C., Jiao S. (2020). Effect of radio frequency heating stress on sublethal injury of *Salmonella* Typhimurium in red pepper powder. LWT Food Sci. Technol..

[32] Zhao W., Yang R., Shen X., Zhang S., Chen X. (2013). Lethal and sublethal injury and kinetics of *Escherichia coli*, *Listeria monocytogenes* and *Staphylococcus aureus* in milk by pulsed electric fields. Food Control.

[33] Miller F.A., Brandão T.R.S., Teixeira P., Silva C.L.M. (2006). Recovery of heat-injured *Listeria innocua*. Int. J. Food Microbiol..

[34] Li L., Mendis N., Trigui H., Oliver J.D., Faucher S.P. (2014). The importance of the viable but non-culturable state in human bacterial pathogens. Front. Microbiol..

[35] Pinto D., Santos M.A., Chabel L. (2013). Thirty years of viable but nonculturable state research: Unsolved molecular mechanisms. Crit. Rev. Microbiol..

[36] Angarano V., Smet C., Akkermans S., Watt C., Chieffi A., Van Impe J.F. (2020). Visible Light as an Antimicrobial Strategy for Inactivation of *Pseudomonas fluorescens* and *Staphylococcus epidermidis* Biofilms. Antibiotics.

[37] Govaert M., Smet C., Baka M., Ećimović B., Walsh J.L., Van Impe J. (2018). Resistance of L. monocytogenes and *S*. Typhimurium towards Cold Atmospheric Plasma as Function of Biofilm Age. Appl. Sci..

[38] Govaert M., Smet C., Vergauwen L., Ećimović B., Walsh J.L., Baka M., Van Impe J. (2019). Influence of plasma characteristics on the efficacy of Cold Atmospheric Plasma (CAP) for inactivation of *Listeria monocytogenes* and *Salmonella* Typhimurium biofilms. Innov. Food Sci. Emerg. Technol..

[39] Govaert M., Smet C., Graeffe A., Walsh J.L., Van Impe J.F.M. (2020). Inactivation of *L. monocytogenes* and S. typhimurium Biofilms by Means of an Air-Based Cold Atmospheric Plasma (CAP) System. Foods.

[40] Smet C., Noriega E., Rosier F., Walsh J.L., Valdramidis V.P., Van Impe J.F. (2016). Influence of food intrinsic factors on the inactivation efficacy of cold atmospheric plasma: Impact of osmotic stress, suboptimal pH and food structure. Innov. Food Sci. Emerg. Technol..

[41] Smet C., Noriega E., Rosier F., Walsh J.L., Valdramidis V.P., Van Impe J.F. (2017). Impact of food model (micro)structure on the microbial inactivation efficacy of cold atmospheric plasma. Int. J. Food Microbiol..

[42] Smet C., Baka M., Steen L., Fraeye I., Walsh J.L., Valdramidis V.P., Van Impe J.F. (2019). Combined effect of cold atmospheric plasma, intrinsic and extrinsic factors on the microbial behavior in/on (food) model systems during storage. Innov. Food Sci. Emerg. Technol..

[43] Verheyen D., Baka M., Van Impe J.F.M. (2019). Sublethal Injury Caused to *Listeria monocytogenes* by Natural Plant Extracts: Case Study on Grape Seed Extract and Garlic Extract. Appl. Sci..

[44] Verheyen D., Baka M., Akkermans S., Skåra T., Van Impe J.F. (2019). Effect of microstructure and initial cell conditions on thermal inactivation kinetics and sublethal injury of Listeria monocytogenes in fish-based food model systems. Food Microbiol..

[45] Verheyen D., Govaert M., Seow T.K., Ruvina J., Mukherjee V., Baka M., Skåra T., Van Impe J.F. (2020). The Complex Effect of Food Matrix Fat Contect on Thermal Inactivation of *Listeria monocytogenes*: Case Study in Emulsion and Gelled Emulsion Model Systems. Front. Microbiol..

[46] Van Impe J.F., Bernaerts K., Geeraerd A.H., Poschet F., Versyck K.J., Tijskens L.M.M., Hertog M.L.A.T.M., Nicolaï B.M. (2001). Modelling and prediction in an uncertain environment. Food Process Modelling.

[47] Walter E., Pronzato L. (1997). Identification of Parameteric Models from Experimental Data.

[48] Perni S., Chalise P.R., Shama G., Kong M.G. (2007). Bacterial cells exposed to nanosecond pulsed electric fields show lethal and sublethal effects. Int. J. Food Microbiol..

[49] Bigelow W.D., Esty J.R. (1920). The thermal death point in relation to time of typical thermophilic organisms. J. Infect. Dis..

[50] Cerf O. (1977). Tailing of survival curves of bacterial spores. J. Appl. Bacteriol..

[51] Akkermans S., Smet C., Valdramidis V., Van Impe J., Demirci A., Feng H., Krishnamurthy K. (2020). Microbial Inactivation Models for Thermal Processing. Food Safety Engineering.

[52] Geeraerd A.H., Herremans C.H., Van Impe J.F. (2000). Structural model requirements to describe microbial inactivation during a mild heat treatment. Int. J. Food Microbiol..

